# Proton-coupled electron transfer at a mis-metalated zinc site detected with protein charge ladders†‡

**DOI:** 10.1039/d4cp01989j

**Published:** 2024-08-28

**Authors:** Mayte Gonzalez, Matthew J. Guberman-Pfeffer, Jordan C. Koone, Chad M. Dashnaw, Travis J. Lato, Bryan F. Shaw

**Affiliations:** a Department of Chemistry and Biochemistry, Baylor University Waco TX USA bryan_shaw@baylor.edu

## Abstract

Distinguishing proton-coupled electron transfer (PCET) from uncoupled electron transfer (ET) in proteins can be challenging. A recent investigation [J. C. Koone, M. Simmang, D. L. Saenger, M. L. Hunsicker-Wang and B. F. Shaw, *J. Am. Chem. Soc.*, **145**, 16488–16497] reported that protein charge ladders and capillary electrophoresis can distinguish between single ET, PCET, and two-proton coupled ET (2PCET) by directly measuring the change in protein net charge upon reduction/oxidation (Δ*Z*_ET_). The current study used similar methods to assess PCET in zinc-free, “double copper” superoxide dismutase-1 (4Cu-SOD1), where one copper is bound at the copper site of each monomer and one copper is bound at the bridging zinc site, resulting in a quasi-type III Cu center. At pH 7.4, the net charge (*Z*) of the 4Cu-SOD1 dimer was unaffected by reduction of all four Cu^2+^ ions, *i.e.*, Δ*Z*_4ET_ = −0.09 ± 0.05 per dimer (−0.02 ± 0.01 per copper atom). These values suggest that PCET is taking place at all four Cu atoms of the homodimer. Molecular dynamics and Poisson–Boltzmann calculations suggest that a metal-coordinating histidine at the zinc site (His71) is the proton acceptor. These data show how ligands of a naturally occurring zinc site can help facilitate PCET when the right redox metal is bound.

## Abstract 2

Distinguishing ET and PCET processes is challenging. This paper used “protein charge ladders” to show that PCET occurs at the non-redox “structural” zinc sight of SOD1, when a Cu ion displaces Zn.

## Graphical abstract

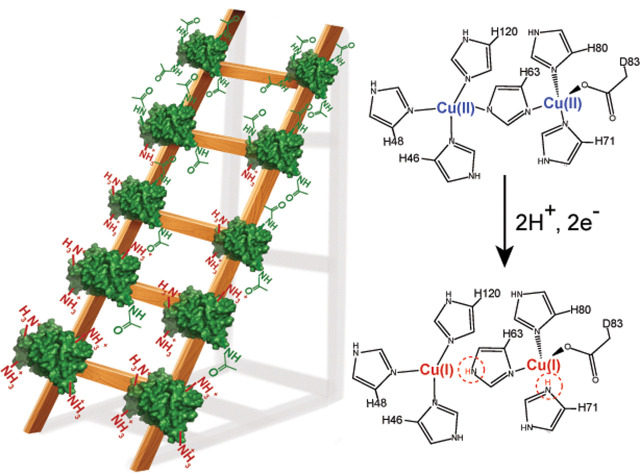

## Introduction

A common method for determining whether an electron transfer (ET) reaction is coupled to proton transfer is to measure the redox potential (*E*) of the reaction as a function of pH.^1–5^ Stoichiometric proton-coupled electron transfer (PCET) reactions will yield a slope of approximately −60 mV pH^−1^ in Pourbaix-style plots of *E vs*. pH.^6–8^ Reactions involving electron transfer without proton coupling will yield horizontal lines. This method is not always suitable for metalloproteins because of pH-dependent conformational changes and the need for chemical mediators.^9–11^ Kinetic methods for detecting PCET also exist.^12,13^

A new method has been recently established for distinguishing ET and PCET in proteins.^14^ This new method uses “protein charge ladders” and capillary electrophoresis (CE) to measure how the protein's net charge (*Z*) fluctuates during redox cycling.^14^ The goal of the current study is to use this new method to determine if PCET can occur at a naturally occurring zinc site when a Cu^2+^ ion is bound at the site (instead of Zn^2+^) and to Cu^1+^.

A protein charge ladder is an electrostatic array of proteins – commonly prepared by acylation of surface lysine residues – that have systematically altered surface charge but similar shape.^15^ Originally developed by Whitesides and co-workers to quantify electrostatic effects in molecular recognition and protein net charge,^16,17^ a protein charge ladder of a metalloprotein can determine its degree of charge regulation upon electron transfer.^14^ When analyzed by CE, a charge ladder can quantify how the net electrostatic charge of the folded, solvated metalloprotein changes with metal oxidation state. Measuring charge regulation in redox reactions can discern whether a redox reaction involves PCET,^18^ ET,^19^ or even two-proton coupled electron transfer (2PCET), where single electron transfer is coupled to the transfer of two protons.^14^

Charge regulation during redox reactions involving metalloproteins can manifest as a net increase in the p*K*_a_ of ionizable residues in response to a reduction in metal oxidation state (and *vice versa*).^18–20^ Charge regulation can explain why the change in net charge (Δ*Z*_ET_) of a metalloprotein upon single electron transfer falls between −1 ≤ Δ*Z* ≤ +1.^19^ Direct measurements of charge regulation during PCET and ET in proteins have been made for heme, Rieske, and type I and II copper metalloproteins using protein charge ladders and capillary electrophoresis.^14,15,18,19^ These studies show that protonation can occur: (i) stoichiometrically at one or two metal binding residues, with no detectable Δ*Z*_ET_ of other residues,^18^ or (ii) sub-stoichiometrically (fractionally) across multiple non-coordinating residues that are distal to the active site.^19^

**Fig. 1:**
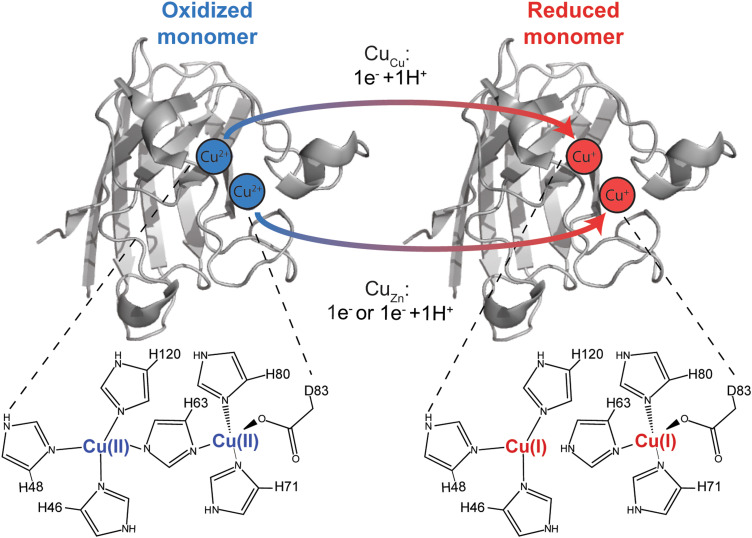
Electron transfer in the Cu, Cu derivative of Cu, Zn SOD1 (denoted Cu_Cu_, Cu_Zn_ SOD1). Proton coupling with electron transfer at the canonical copper site is well established (His63 becomes protonated upon reduction of Cu^2+^ to Cu^1+^). Is a proton also coupled with electron transfer to copper bound at the canonical “structural” zinc site (Cu_Zn_)?

For example, in the [2Fe–2S] Rieske protein, protonation and ET appear to be perfectly stoichiometric; upon reduction of one Fe^3+^ to Fe^2+^, proton transfer occurs at zero, one, or two histidine residues as solvent pH increases from 5 to 10.^14^ Thus, at high pH, a Rieske protein can actually become more positively charged upon reduction of Fe^3+^, a consequence of 2PCET. With human Cu, Zn superoxide dismutase (hSOD1), PCET is also stoichiometric, involving proton transfer to a histidine that bridges Cu and Zn (His63), with no net change in protonation of other residues (Fig. 1).^18^ In the type I copper protein azurin, single electron transfer is coupled (to some degree) to the partial protonation of a handful of non-coordinating residues that are distal to the active site.^19^ It has been noted that the free energy associated with the protonation of these residues in azurin could account for a large fraction of the total reorganization energy measured by Gray and co-workers.^21^ This type of fractional, sub-stoichiometric protonation is also observed with myoglobin.^19^

## Materials and methods

### Purification and characterization of SOD1

Wild-type (WT) superoxide dismutase 1 (SOD1) was recombinantly overexpressed in *S. cerevisiae*. SOD1 was purified as previously reported.^22,23^ Yeast stocks were made by transfecting Yep351-hSOD1 plasmids into EG118Δsod1 yeast. This was then followed by growing primary cultures to OD_600nm_ ∼ 1.5 (approximately 36 hours) in YPD media and transferring into larger cultures for ∼7 days. The grown cells were spun down, lysed, and purified through ammonium sulfate precipitation followed by three chromatographic separations: hydrophobic interaction chromatography, ion exchange chromatography, and size exclusion chromatography. SOD1 purity was confirmed *via* SDS-PAGE following each chromatographic separation, and the final protein solution was characterized by mass spectrometry.

### Demetalation of Cu, Zn SOD1

Purified SOD1 solutions were demetalated *via* sequential dialysis over 6 days in: (i) 100 mM ammonium acetate, 5 mM EDTA, pH 3.8; (ii) 100 mM ammonium acetate, 100 mM NaCl, pH 3.8; and (iii) 100 mM ammonium acetate, pH 5.5. The protein was in each buffer for ∼2 days with buffer replacement every ∼8 hours. To prevent metal contamination, all glassware was rinsed with 10 mM EDTA, followed by Milli-Q water. Inductively coupled plasma-mass spectrometry (7900 ICP-MS, Agilent Technologies) was used to verify full demetalation of SOD1 to be <0.08 equivalents of copper and zinc (Table S1, ESI‡).

### Acetylation of apo-SOD1 to yield a “protein charge ladder”

The apo-SOD1 was acetylated with acetic anhydride as previously described to generate protein charge ladders.^24^ Briefly, the protein was washed into 100 mM HEPBS (pH 9) *via* centrifugal filtration to a final concentration of ∼150 μM. 100 mM acetic anhydride was made in 1,4-dioxane. Three molar equivalents of acetic anhydride were added to the protein solutions to generate a protein charge ladder. Extent of acetylation of SOD1 was confirmed through electrospray ionization-mass spectrometry (ESI-MS) using a ThermoFisher™ Discovery Orbitrap mass spectrometer. Protein samples were prepared by diluting with 0.2% formic acid and removing salt with a desalting column (Michrom BioResources, Inc., Auburn CA, USA) followed by eluting with 80% acetonitrile, 20% of 2% formic acid. Acetylated SOD1 was transferred into the appropriate running buffer for CE experiments through centrifugal filtration (4000 × *g* at 4 °C).

### Remetalation of apo-SOD1 to 4Cu-SOD1 and reduction with sodium dithionite

Acetylated apo-SOD1 WT (*i.e.*, the apo-SOD1 “protein charge ladder”) was titrated slowly with up to five molar equivalents of 50 mM of CuSO_4_ over 30 minutes. Solutions were incubated at 4 °C for a period of 2 days with metal binding and metal content verified with UV-vis and ICP-MS (Table S1, ESI‡). Protein charge ladders of reduced 4Cu-SOD1 were prepared by addition of 160 molar equivalents of sodium dithionite to the oxidized Cu–Cu SOD1 protein charge ladder.

### Capillary electrophoresis

All CE experiments were performed using a Beckman P/ACE MDQ instrument with a bare fused-silica capillary. Electrophoresis was performed at 29 kV in corresponding buffers and the capillary was cooled to 22 °C to prevent Joule heating. The concentration of SOD1 protein charge ladders analyzed with CE was 30 μM SOD1 dimer. Dimethylformamide (DMF) was added as a neutral marker of electroosmotic flow (EOF). Capillary conditioning was performed in between experiments with washes of 0.1 M HCl (2 min), methanol (2 min), 0.1 M KOH (2 min), Milli-Q water (2 min), and running buffer (4 min). All protein concentrations were determined *via* UV-vis spectrometry *ε*_280_: 10 800 cm^−1^ M^−1^ for apo-SOD1 and *ε*_265_: 18 400 cm^−1^ M^−1^ holo-SOD1. For experiments involving protein charge ladders of reduced Cu, Cu SOD1, the running buffer used was deoxygenated *via* nitrogen bubbling for at least 3 hours prior to electrophoresis. All reduced protein solutions were analyzed with CE immediately following the addition of sodium dithionite.

Electrophoretic mobility was calculated by using eqn (1) to convert the migration times of unacetylated SOD1 and each rung of the charge ladder for each electropherogram.1
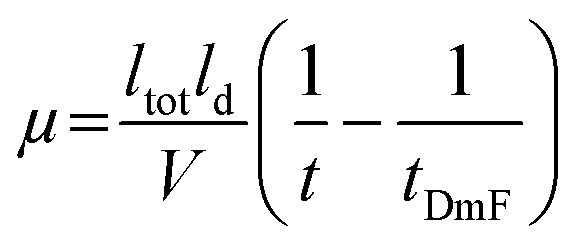
The equation represents the mobility of protein species defined by *μ*.

### Computational methods overview

Nearly 3 μs of production-stage classical molecular dynamics, with and without variable protonation of titratable residues, and hundreds of Poisson–Boltzmann electrostatic energy calculations were performed for homodimeric Cu_Cu_Cu_Zn_-SOD1. The simulations comprised two stages: (1) evaluating the change in p*K*_a_ for titratable residues in the second coordination sphere upon reduction of Cu_Zn_, (at this stage, the Cu was assumed to retain the H63, H71, H80, and D83 ligands in both redox states); and (2) assessing the energetic change upon protonating each of the four ligands to the reduced Cu in the Zn-binding site. All simulations were performed assuming the Cu in the Cu site was already reduced, and this Cu had lost coordination to both a water molecule and His63.

### Metal site parameterization

A prerequisite for simulations of 4Cu-SOD1 was the derivation of molecular mechanics parameters for Cu_Cu_^1+^, Cu_Zn_^2+^, and Cu_Zn_^1+^. The parameters were derived with the assistance of the Metal Center Parameter Builder (MCPB)^25^ of the AmberTools22 Suite.^26,27^ Bonded parameters were obtained with the Seminario method^28^ from quantum mechanically (QM) optimized models including the Cu ion and coordinating ligands up-to-and-including the β-carbons. The QM optimization and frequency calculations were performed with the Becke, three-parameter, Lee–Yang–Parr (B3LYP) approximate density functional^29,30^ and a mixed basis set (LANL2DZ for Cu;^31^ 6-31G(d) for H, C, N and O),^32–34^ as implemented in Gaussian 16 Rev. A.03.^35^ Merz–Singh–Kollman atomic partial charges for the Cu ion and coordinating ligands were obtained with the aforementioned model chemistry and the restrained electro-static potential (RESP) approach. Backbone (heavy and hydrogen) atoms of the coordinating ligands were held to the standard force field values (option 3c in MCPB). The metal center parameters were developed for use with the ff10 AMBER forcefield^36^ to be compatible with constant pH molecular dynamics simulations. The library and forcefield modification (frcmod) files needed for the simulations are provided as ESI‡ at the following GitHub repository: https://github.com/Mag14011/CuCuSOD1_SI_CompMethods.

### Structure preparation

A dimer (chains A and F) of the highest (1.07 Å) resolution X-ray crystallographic structure for Cu–Zn human superoxide dismutase 1 (SOD1) available in the Protein Data Bank (accession code 2C9V)^37^ was selected as the starting point for the modeling in the present work. Preparatory steps included: (1) replacing Zn with Cu in the canonical Zn binding site, (2) replacing chain F with a superimposed copy of chain A (because there is a >3 Å break in chain F), (3) acetylating the N-terminus of each chain, (4) selecting the highest occupancy position when alternate coordinates were present, except for the Cu ion in the Cu site for which the low occupancy position corresponded to the reduced state, and (5) changing the residue names of metal-center ligands to match the above-described parametrization, and (6) changing the names of second coordination sphere ASP, GLU, and HIS residues to ASH, GLH, and HIP so these residues (as well as TYR and LYS for which a name change was not needed) can be titrated in constant pH molecular dynamics (CpHMD).

4Cu-SOD1 was simulated in the Cu^1+^–Cu^2+^ and Cu^1+^–Cu^1+^ redox states as a function of pH from −4 to 12 in increments of 2 pH units. 4Cu-SOD1 was also simulated in the Cu^1+^–Cu^1+^ state with each ligand (His63, His71, His80, and Asp83) to the Cu ion in the canonical Zn site either unprotonated or protonated. For the unprotonated cases, the bonded parameters between the ligand and Cu ion were deleted, but the electrostatic interaction was retained, essentially adopting a non-bonded or crystal-field-theory approximation. This procedure was necessary because it is not possible to simulate, at the classical mechanics level, the breaking of the coordination bond as the ligand becomes protonated.

tLEaP in the AmberTools22 package was used to add hydrogen atoms to the various SOD1 models. Each structure was placed at the center of a box of explicit water with at least a 15 Å buffer region to the boundary of the box. A sufficient number of counterions (6 and 8 Na^+^, respectively, for the Cu^1+^–Cu^2+^ and Cu^1+^–Cu^1+^ states) was added to achieve charge neutrality. The TIP3P water model^38^ and the monovalent ion parameters of Joung and Cheatham^39^ were used to model the solution state.

### Molecular dynamics

Each solvated structure was subjected to 10 000 steps of steepest descent followed by 40 000 steps of conjugate-gradient minimization, all with a 10 kcal (mol Å^2^)^−1^ restraint on the heavy atoms of the protein backbone. Each system was subsequently heated in the NVT ensemble from 0 to 300 K at a rate of 0.3 K ps^−1^ and held at the final temperature for 3.0 ns. A 1.0 kcal mol^−1^ Å^−2^ restraint on the protein backbone was applied during the heating process and first 1.0 ns at the final temperature, but then reduced to 0.1 kcal mol^−1^ Å^−2^ for the remaining 2.0 ns. After thermalization, the density of each system was equilibrated under 1.0-bar pressure for 2.0 ns in the NPT ensemble. Production stage simulations were conducted in the NVT ensemble at 300 K. The lengths of the various production simulations are shown in (Table S3, ESI‡).

All NVT and NPT simulations (including the production-stage) employed periodic boundary conditions, the particle mesh Ewald^40^ treatment of electrostatic interactions with a direct sum cut-off of 10.0 Å, the SHAKE algorithm^41,42^ to rigidify bonds to hydrogen atoms, a Langevin thermostat with a collision frequency of 2 ps^−1^, and an integration timestep for the Langevin equation of motion of 2.0 fs. Pressure in NPT simulations was regulated with a Monte Carlo barostat having a relaxation time of 1.0 ps. PMEMD in its CPU and GPU^43^ implementations of the Amber22 package^27^ were used to perform the minimization and dynamical simulations, respectively.

### Poisson Boltzmann electrostatic calculations

Configurations from the last 100 ns of each simulation in which a ligand to Cu^1+^ in the canonical Zn site was unprotonated or protonated were selected, stripped of the explicit water and ions, and submitted to the Poisson–Boltzmann surface area (PBSA) program of the AmberTools22 suite. The configurations were sampled every 1.0 ns, giving a total of 100 analyzed frames. The statistics of the total energy before and after protonating a given ligand were compared to assess which ligand is most likely to become protonated when the Cu in the Zn site is in the reduced (1+) state. The BASH script used for this post-simulation analysis is provided as ESI‡ in the following GitHub repository: https://github.com/Mag14011/CuCuSOD1_SI_CompMethods.

## Results and discussion

**Fig. 2:**
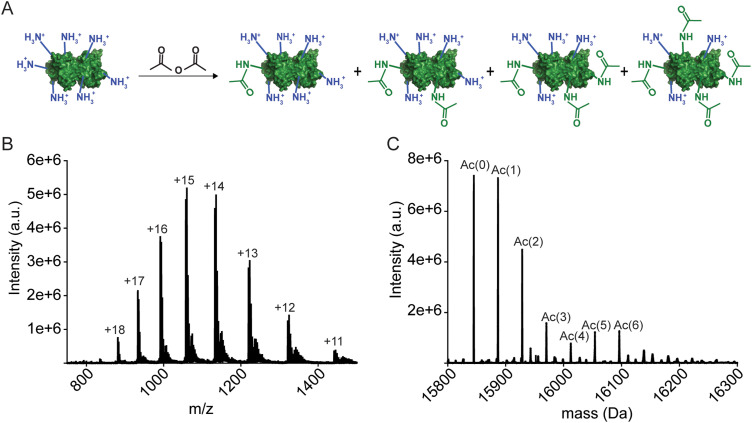
(A) Lysine-acetyl “protein charge ladders” of human wild type SOD1 synthesized by acetylation of surface lysine with acetic anhydride. (B) and (C) Raw electrospray ionization mass spectrum (B) and deconvoluted spectrum (C) for WT apo-SOD1 charge ladders. Note: the SOD1 homodimer dissociates under these ionization conditions (but remain dimeric during capillary electrophoresis).

In this paper, a pseudo-type III copper center was generated in homodimeric Cu, Zn-SOD1 by removing Zn^2+^ from the Zn site of each monomer and replacing it with Cu^2+^ (Fig. 1). This Cu–Cu derivative is denoted Cu_Cu_Cu_Zn_-SOD1 or 4Cu-SOD1.^44–46^ A lysine-acetyl protein charge ladder of this pseudo-type III copper derivative of SOD1 was created (Fig. 2) to determine whether it undergoes 2PCET, ET, or a mixture of the two processes upon reduction of all four Cu^2+^ ions in the 4Cu-SOD1 homodimer. Our previous studies of PCET in metalloproteins (with protein charge ladders) investigated the extent of PCET at naturally occurring redox sites that are properly metalated with redox active metals.^14,18,19^ In contrast, the current study uses protein charge ladders to determine whether PCET occurs at a “mis-metalated” non-redox active site (the zinc site of SOD1) when a redox active metal (copper) has displaced the natural metal (zinc).

Copper centers can typically be classified by stoichiometry, ligand type, and geometry of the metal site.^47^ Type III Cu centers are characterized by: (i) two copper ions (usually separated by ∼3.5 Å with a bridged substrate), (ii) a silent spectroscopic signature due to antiferromagnetic coupling,^48,49^ and (iii) coordination of both copper atoms by three histidine residues. In general, naturally occurring type III Cu proteins (*e.g.*, multicopper oxidases) can perform twin PCET *per se* (*i.e.*, PCET at both copper ions) by coupling proton transfer to an electron through bridging dioxygen substrates that become oxidized.^50,51^

The properly metalated Cu, Zn SOD1 protein is not a type III copper protein. Rather, it contains a unique type II copper center that shares a bridging histidine with a zinc atom.^52^ Type II copper centers consist of a single copper atom coordinated by four ligands (N or N/O) in a square planar geometry. The copper site in SOD1 is five coordinate in the Cu^2+^ oxidation state, coordinated by four histidine residues (one of which, His63, bridges both Cu and Zn sites) and one H_2_O (or O_2_^−^ substrate). This copper site adopts a distorted square pyramid configuration (Fig. 1).^49^ The copper center transitions to a three coordinate distorted trigonal plane upon reduction – which involves PCET – wherein the bridging His63 is protonated upon reduction (Fig. 1).^53^ The zinc site contains three histidine and one aspartate that adopt a nearly tetrahedral coordination geometry when zinc is bound.^54^

The 4Cu-SOD1 derivative was prepared by removing all coordinated metal ions from the recombinant as-isolated human wild-type (WT) SOD1 dimer (using EDTA and pH 3.8 buffer). The removal of Cu and Zn was verified with inductively coupled plasma-mass spectrometry (ICP-MS; Table S1, ESI‡). Protein charge ladders of apo-SOD1 were then formed by acetylating its lysine residues with acetic anhydride, prior to remetalation with copper. Each acetylation increases the mass by 42 Da (Fig. 2A–C). Acetylation of ∼3 lysine residues on each SOD1 subunit (6 lysine per dimer) has a negligible effect on the structure of SOD1, according to previous analysis with hydrogen/deuterium exchange.^55^ It is important to remember that the SOD1 dimer dissociates during ESI-MS. The number of acetylated lysine inferred from the mass spectra correspond to each SOD1 monomer. Note that the recombinant SOD1 protein was expressed in *S. cerevisiae*, *i.e.*, the N-terminus is also properly acetylated.

**Fig. 3:**
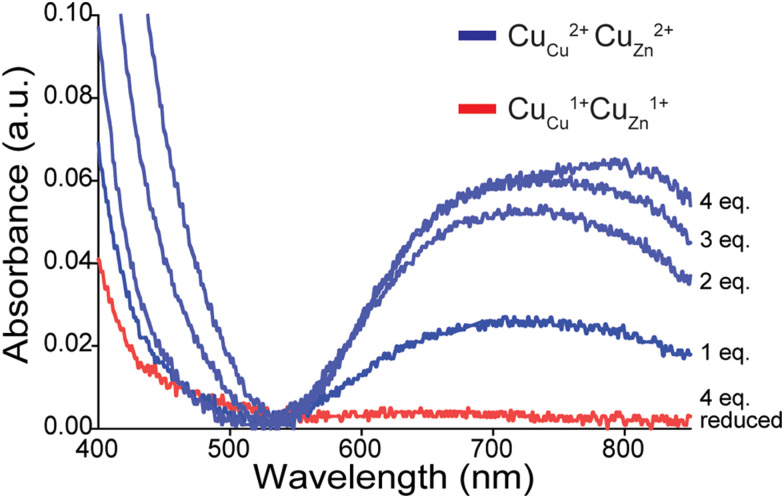
UV-vis spectra of d–d band in a lysine-acetyl protein charge ladder of human WT SOD1 as four stoichiometric equivalents of Cu^2+^ (per dimer) are titrated into the metal-free homodimeric charge ladder over 48 h, pH 7.4 (blue traces). The red spectrum is of the copper-replete 4Cu-SOD1 protein after reduction with 160 stoichiometric equivalents of sodium dithionite (per SOD1 dimer).

After acetylation of apo-SOD1 to form a lysine-acetyl protein charge ladder, four Cu^2+^ (per dimer) were titrated back into the lysine-acetyl charge ladder of apo-SOD1 dimer (Fig. 3), *i.e.*, copper was added after lysine acetylation. Each metal binding site of the 4Cu-SOD1 derivative was occupied by copper, according to UV-vis spectroscopy (Fig. 3). Here, the sequential binding of Cu^2+^ to the SOD1 active site can be observed by the copper d–d transition at 500–800 nm (Fig. 3). Four molar equivalents of Cu^2+^ (per dimer) were added sequentially (1 eq. at a time) and monitored *via* UV-vis to ensure that Cu^2+^ bound to the active site (Fig. 3). Here, the expected increase in the d–d region was observed, indicating coordination Cu^2+^ (Fig. 3). ICP-MS verified copper stoichiometry (Table S1, ESI‡).

CE and protein charge ladders were used to directly measure the net charge (*Z*) of 4Cu-SOD1 in both the oxidized Cu^2+^ and reduced Cu^1+^ state at multiple pH values.^15,18,19,56^ To ensure the maintenance of full copper metalation during CE, the solution of 4Cu-SOD1 protein charge ladder contained an extra molar equivalent of Cu^2+^ (*i.e.*, five molar equivalents were added per dimer).

**Fig. 4:**
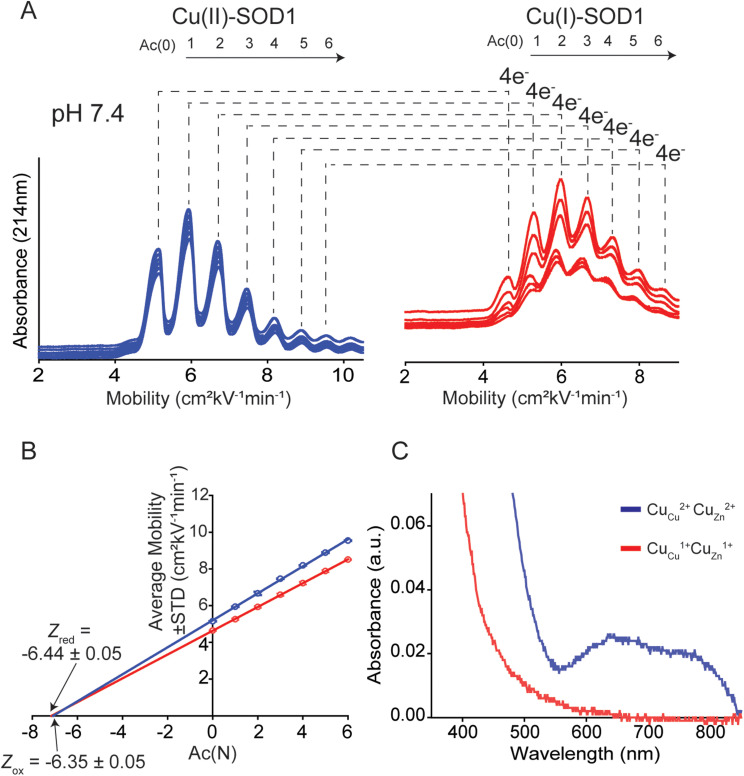
Use of capillary electrophoresis (CE) to measure change in net charge (Δ*Z*_ET_) of Cu–Cu SOD1 upon reduction of all four Cu^2+^ ions. (A) Electropherograms of protein charge ladders of oxidized (blue) and reduced (red) Cu_Cu_Cu_Zn_ SOD1 at pH 7.4 (*i.e.* Cu^2+^, Cu^2+^ and Cu^1+^, Cu^1+^) are shown as overlays of 6 technical replicates. (B) Plot of the electrophoretic mobility (*μ*) of each rung *versus* the number of acetylated lysine(s) Ac(N). The *x*-intercept is equal to the quotient of the net charge (*Z*) of the unacetylated protein and the change in charge with each acetylation (*i.e.*, *Z*_Ac(0)_/Δ*Z*_Ac_). The charge of the reduced and oxidized proteins are indicated by *Z*_red_ and *Z*_ox_. (C) UV-vis spectra of a lysine-acetyl protein ladder of Cu_Cu_Cu_Zn_ SOD1 used for CE analysis, before and after reduction of all four Cu^2+^ in the homodimer to Cu^1+^ using sodium dithionite and deoxygenated buffer.

When separated by CE, a distribution of peaks (rungs) is observed for oxidized 4Cu-SOD1 (Fig. 4A). As expected, the electropherogram of the charge ladder shows a different distribution of peaks than the electrospray ionization mass spectrum. This difference is observed because electrospray ionization monomerizes SOD1 (complete dissociation of the SOD1 dimer), whereas the SOD1 dimer remains intact during CE. Thus, the third rung of the ladder (“Ac 2”, a dimer with two acetylations) can be comprised of either one monomer with zero acetylations bound to a monomer with two acetylation, or two monomers bound together, each with one acetylation.

The *Z* of the unmodified protein is determined by extrapolating the linear trend of electrophoretic mobility (*μ*) and acetylation number Ac(N) (Fig. 4B). In this paper, the first five to six rungs of each charge ladder were used to determine the *Z* of oxidized and reduced 4Cu-SOD1. CE only requires nanoliters of sample and has a run time of less than 15 minutes, which allows collection of multiple replicates (>6 technical replicates) to yield statistically significant values.^18,19^

### The net charge of 4Cu-SOD1 does not change upon reduction at physiological pH

At pH 7.4, the net charge of the oxidized 4Cu-SOD1 dimer was measured to be *Z* = −6.35 ± 0.05 (Fig. 4A and B). This value differs slightly from a value measured previously for the properly metalated Cu, Zn SOD1 dimer (*i.e.*, *Z* = −7.32 ± 0.17).^18^ This difference might be due to actual differences in net charge of the Cu–Cu and Cu–Zn SOD1 proteins (due to different residue p*K*_a_, or coordinated solvent/co-solvent ions) or it might be caused by the different buffer conditions. In the current study, 50 mM 3-morpholinopropane-1-sulfonic acid buffer (MOPS) was used, whereas potassium phosphate was used in prior studies.^15^

The reduction of all Cu^2+^ ions in the copper loaded SOD1 was accomplished by the addition of 160 equivalents of sodium dithionite (Fig. 4A). Upon complete reduction, *Z* changed from −6.35 ± 0.05 to −6.44 ± 0.05, *i.e.*, Δ*Z* of −0.09 ± 0.05 per dimer or −0.02 per copper (Fig. 4B). This value suggests that 4Cu-SOD1 undergoes complete charge regulation at pH 7.4 upon reduction of all four copper ions. Previous studies have confirmed – as does this one – that both copper atoms in each subunit can be reduced with dithionite.^14,18,19^ UV-vis spectra were obtained for the oxidized SOD1 samples prior to CE (Fig. 4C).^18,57^

### Charge regulation in 4Cu-SOD1 is minimally affected by pH

Charge was also measured at pH 5.6 and 8.3 (the physiologically relevant pH range of SOD1) to assess effects of pH on charge regulation in 4Cu-SOD1.

**Fig. 5:**
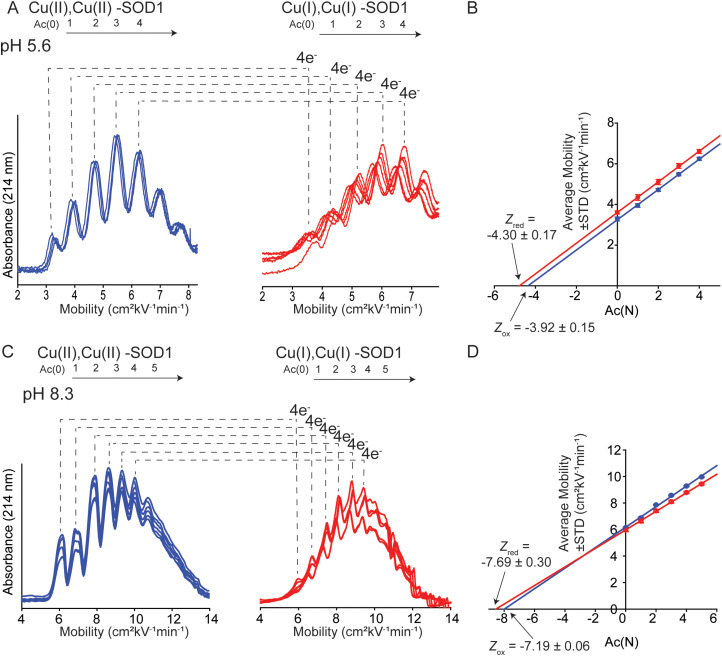
(A) Capillary electropherograms of protein charge ladders of oxidized (blue) and reduced (red) Cu_Cu_Cu_Zn_ SOD1 at pH 5.6 are shown as overlays of 6 technical replicates. (B) The plot of the electrophoretic mobility (*μ*) of each rung *versus* the number of acetylated lysine Ac(N) at pH 5.6. (C) Electropherograms of protein charge ladders of oxidized (blue) and reduced (red) of Cu_Cu_Cu_Zn_ SOD1 at pH 8.3 are shown as overlays of 6 technical replicates. (D) The plot of mobility of each rung *versus* Ac(N) for each rung at pH 8.3. Values of the charge of the reduced and oxidized proteins are indicated by *Z*_red_ and *Z*_ox_.

Electrophoresis was performed at pH 5.6 in 10 mM sodium acetate (Fig. 5A). Analysis of the electropherograms showed that the *Z* of 4Cu-SOD1 was −3.92 ± 0.15 per dimer, and −4.30 ± 0.17 after reduction with sodium dithionite (Δ*Z*_4ET_ = −0.38 ± 0.23 or Δ*Z*_ET_ = −0.09 ± 0.23 per electron) (Fig. 5B). The data suggests that charge regulation weakened (*i.e.*, magnitude of Δ*Z* became larger) but persisted at lower pH. The percent of charge regulation per electron was 91%, 7% less than the charge regulation observed at pH 7.4. The lower resolution of the electropherograms at pH 5.6 is caused (in part) by the slower electroosmotic flow from protonation of silanol groups (SiOH) in the capillary, making measurements at pH ≤ 5 difficult with CE (Fig. 5A).

For experiments at pH 8.3, CE experiments were performed in 20 mM Tris-Gly (Fig. 5C). Charge regulation was not as pronounced at pH 8.3, with Δ*Z*_4ET_ = −0.51 ± 0.31 (Δ*Z*_ET_ = −0.13 ± 0.14 per electron) (Fig. 5D). The percent of charge regulation per electron was 87% (*i.e.*, 11% less than the charge regulation taking place at pH 7.4 and 4% less than at pH 5.6) (Table 1). These values suggest that PCET is occurring at pH 8.3.

**Table 1:** The measured net charge (*Z*_CE_) of hSOD1, and the change in net charge upon reduction (Δ*Z*_ET_) of all four Cu^2+^ ions in each dimer

Metal redox state	pH	*Z*_CE_ a	Δ*Z*^a^ (*Z*_ox_ − *Z*_red_)	Δ*Z* per electron	% Charge regulation per electron
Cu^2+^	5.6	−3.92 ± 0.15	−0.38 ± 0.23	−0.09 ± 0.23	91 ± 6
Cu^1+^	5.6	−4.30 ± 0.17			
Cu^2+^	7.4	−6.35 ± 0.05	−0.09 ± 0.05	−0.02 ± 0.05	98 ± 1
Cu^1+^	7.4	−6.44 ± 0.05			
Cu^2+^	8.3	−7.19 ± 0.06	−0.50 ± 0.14	−0.13 ± 0.14	87 ± 4
Cu^1+^	8.3	−7.69 ± 0.30			

a Values are listed per dimer.

Note that electropherograms for reduced 4Cu-SOD1 proteins (Fig. 4 and 5) appear to exhibit slightly lower resolution compared to electropherograms of oxidized proteins. In general, peak resolution in CE can be dependent on three major factors: (i) the length of the sample “plug”; (ii) diffusion between the time of injection and detection; and (iii) interactions between the protein and the surface of the fused silica capillary.^58–60^ Adhesion of protein to the capillary is the likely cause of the lower resolution of reduced SOD1.

It is also important to comment on the error associated with replicate electropherograms of some of the protein charge ladders. There is, for example, larger variation in mobility values for reduced charge ladders in Fig. 5A compared to Fig. 4A. The variable mobility values in Fig. 5A do not lead to large variations in the final, calculated net charge. The data set of electropherograms in Fig. 4A (copper-reduced) consists of nearly superimposed electropherograms. Here the mobility of rung 2 (*i.e.*, Ac(2)) varied from *μ* = 5.87 to 5.98 cm^2^ kV^−1^ min^−1^ across replicate measurements. This variation results in a small variation in *Z*, *i.e.*, *Z* = −6.52 to −6.43. In contrast, the copper-reduced electropherograms of Fig. 5A are more variable. Here, rung 2 varied from *μ* = 4.93 to 5.27 cm^2^ kV^−1^ min^−1^. This results in larger (but still small) variation in *Z*, *i.e.*, from *Z* = −4.26 to −4.51.

### PCET during Cu_Zn_ reduction: where is the proton going?

Our previous report found that properly metalated SOD1 (Cu_2_Zn_2_-SOD1) undergoes complete charge regulation upon ET.^18^ This complete charge regulation was due to PCET *via* protonation of a bridging histidine upon reduction of Cu^2+^.^61,62^ We expected the value for Δ*Z*_ET_ of 4Cu-SOD1 to be approximately −2 per dimer, accounting for single ET at the two Cu_Zn_ and PCET at each Cu_Cu_. That is, the Cu_Zn_ site was not expected to participate in PCET but rather single, uncoupled ET.

Classic electrochemical studies of SOD1 support the hypothesis that Cu^2+^ bound to the zinc site engages in PCET.^3^ For example, the Pourbaix diagram of a copper SOD1 derivative where the zinc site of each monomer is empty (Cu_2_E_2_ SOD1) suggests proton coupling is occurring at pH > 7,^3^*i.e.*, a pH range where Cu is known to migrate from the copper site to the empty zinc site.^63^ The slope of mV pH^−1^ at pH > 7 for this Cu_Zn_ derivative is, however, only −18 mV pH^−1^.^3^

The likely occurrence of PCET at the zinc site raises the question: which amino acid residues are being protonated during reduction of Cu_Zn_? Is protonation occurring on one residue, or multiple residues? Are these residues at, near, or distal to the Zn site?

**Fig. 6:**
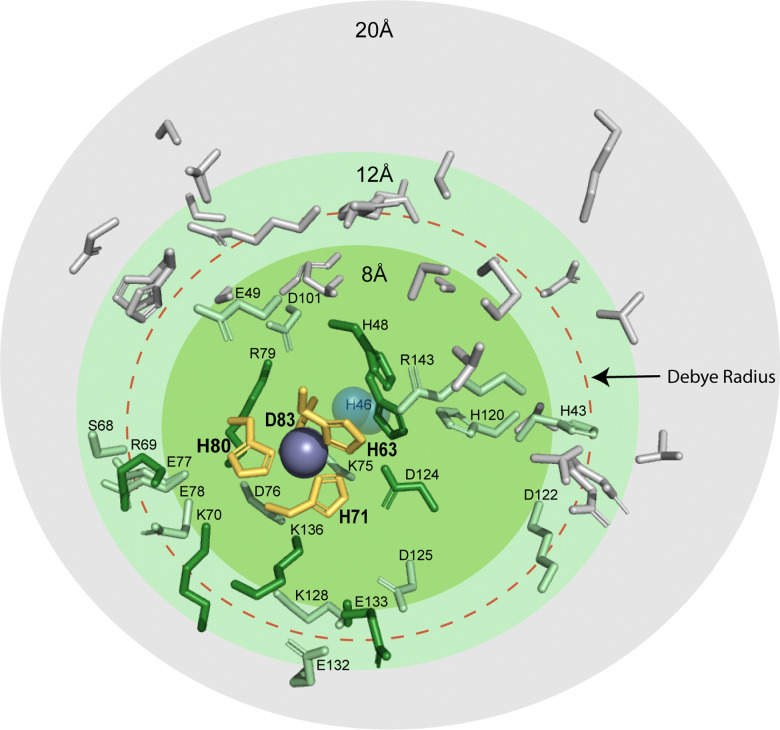
Protic residues within 20 Å of the zinc site in SOD1 that might be potential proton acceptors during electron transfer (PDB: 2C9V). The residues that coordinate metals in the zinc site are shown in yellow (H63, H71, H80, and D83). Ionizable residues are shown within 8 Å (green), 12 Å (light green), and 20 Å (grey). The red dashed circle denotes the classical Debye radius in physiological buffer (10 Å, but which can be much longer in hydrophobic interiors).

The residue responsible for protonation during PCET of Cu_Cu_ in SOD1, His63, is thought to exist as a doubly deprotonated imidazolate.^64,65^ There are several possible sites that could participate in PCET at Cu_Zn_: (i) the bridging H63 imidazolate, which would become doubly protonated, (ii) a Cu_Zn_ coordinated residue becoming protonated (H71, H80, or D83 might become protonated), or (iii) residue(s) distal to the active site becoming protonated as electrostatic interactions span ∼10 Å in physiological conditions and are longer in hydrophobic environments. Distal ionizable residues surrounding the Cu_Zn_ site might adjust p*K*_a_.^15^ Within 20 Å of the Cu_Zn_ there are a total of 46 ionizable residues which may contribute to diffuse charge regulation following ET (Fig. 6). There are 9 residues located from 4 Å to 10 Å (H46, K70, R79, D124, K136, E133, H48, R69, S134). Here, there is 1 located within 4 Å (K136), 2 within 5 Å (H46, K136), and 5 within 6 Å (H46, K70, R79, D124, K136). These distal ionizable residues may be located near the active site to participate in diffuse charge regulation, where p*K*_a_ values of multiple residues change.

**Fig. 7:**
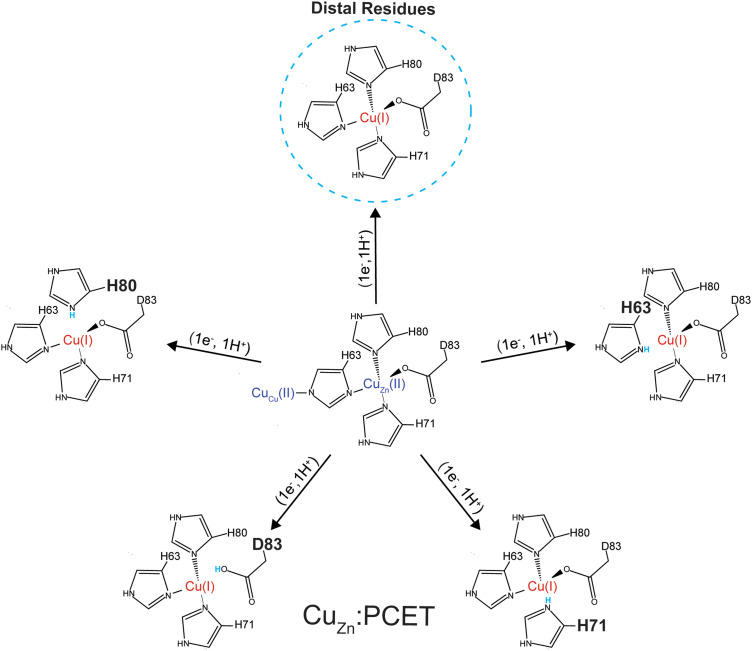
Five possible sites of protonation during electron transfer to Cu^2+^ bound in the zinc site of SOD1. Four sites include: H80, D83, H71, H63 and a fifth “site” includes one or more residues in the second coordination sphere (*e.g.*, D76, D124, or D125) or residues that are more distal to the metal site.

The most reasonable hypothesis is that protonation is taking place at the Cu_Zn_ coordinating ligands (H71, H80, D83) (Fig. 7). Since the reduction of Cu^2+^ to Cu^1+^ induces a change in coordination geometry (*i.e.*, tetrahedral to trigonal planar), theoretically, it is possible that H71 could become uncoordinated from Cu(i) where it could participate in a hydrogen bonding network that involves D124.^66^

Determining which residues are participating in PCET at the Cu_Zn_ site can be experimentally challenging. Therefore, a two-stage computational approach based on classical molecular dynamics simulations to make an approximation of which residues are becoming protonated upon reduction of Cu_Zn_ was conducted.

First, the change in p*K*_a_ upon reduction of the Cu^2+^ ion in the Zn binding site was assessed for the 10 Asp, 10 Glu, 2 His, and 11 Lys residues in the second coordination sphere of each subunit of 4Cu-SOD1 (Table S2, ESI‡). From this analysis only four residues were predicted to exhibit p*K*_a_ changes ≥1.0 unit: D101 (1.4 in one subunit, but 0.7 in the other), D124 (2.6–2.9 units in both subunits), and D125 (1.0 in one subunit, but −0.4 in the other). Furthermore, the p*K*_a_'s of all these residues remained at least 2 units below the lowest pH examined experimentally, thereby suggesting that second-sphere titratable residues are not the site of charge regulation (protonation) upon reduction of Cu_Zn_.

Given this null result, the second stage of computations involved assessing the energetics of protonating each of the four primary coordination sphere ligands to the Cu_Zn_ site: H63, H71, H80, and D83. Dynamical trajectories were performed in the fully reduced state for 4Cu-SOD1 with only three of these four residues explicitly bonded to the Cu^1+^ ion in the Zn binding site; the fourth possible ligand was simulated as either unprotonated or protonated. Since it is not possible to simulate the breaking of a ligand–metal bond in a classical mechanical simulation, we adopted the crystal field theory approximation that the ligand–metal interaction was entirely electrostatic in nature. This assumption allowed us to ask the question: is it electrostatically more favorable for one of these four ligands to interact with the Cu^1+^ center or to become protonated?

**Fig. 8:**
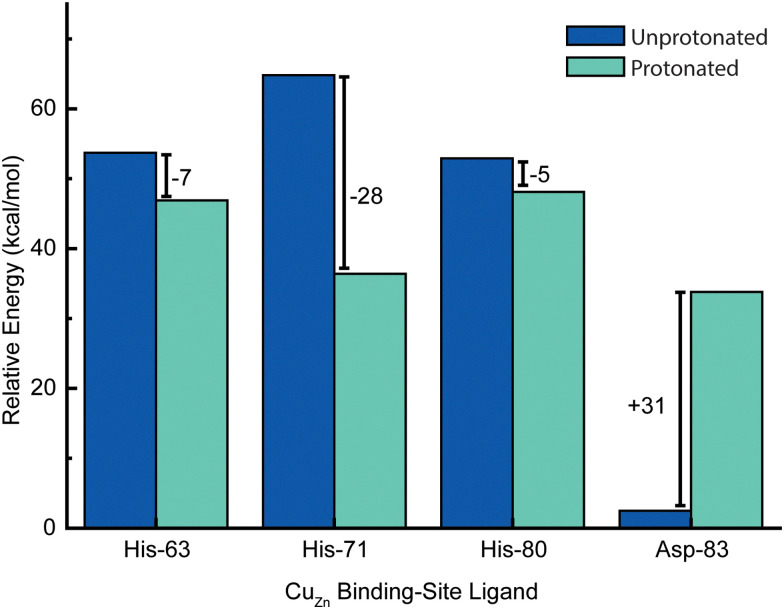
Change in relative energies of copper ligands (of the zinc site) upon protonation in the Cu^1+^ oxidation state. In this simulation, both Cu_Zn_ and Cu_Cu_ are in the +1 oxidation state. Energies represent computed averages over thermal ensembles of configurations sampled during molecular dynamics trajectories and assessed with Poisson–Boltzmann calculations with the protein explicitly included.

Energetic assessments of configurations from these trajectories with the Poisson–Boltzmann surface area (PBSA) method gave the following rank ordering of the four primary coordination sphere residues in terms of increasing stability upon protonation when Cu_Zn_ is reduced: D83 (+31 kcal mol^−1^) < H80 (−4 kcal mol^−1^) ≈ H63 (−6 kcal mol^−1^) < H71 (−28 kcal mol^−1^) (Fig. 8). We therefore propose that H71 is the site of proton uptake upon reduction of the Cu in the Zn binding site of SOD1.

## Conclusion

This study has shown that a naturally occurring non-redox “structural” metal site – the zinc site of Cu, Zn SOD1 – has the necessary structural and chemical elements to undergo PCET when a redox active metal (copper) is bound. This near zero value of Δ*Z*_ET_ for this site suggests that the entire protein framework of the 4Cu-SOD1 dimer is insensitive to the redox state of its four coordinated copper ions. It is possible that the four protons that are coupled to ET in the copper replete homodimer might act as electrostatic shields, to prevent distal non-coordinating ionizable residues within the Debye radius from having p*K*_a_ shifts upon ET. This insensitivity of net charge to metal oxidation state might be important for the diffusion-limited reduction and oxidation of superoxide radicals in the SOD1 protein.

**Fig. 9:**
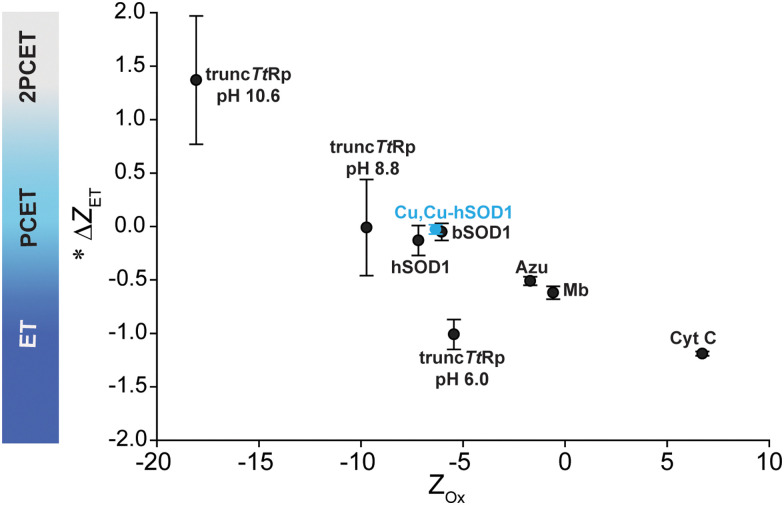
Plot of *Z*_ox_*vs*. Δ*Z*_ET_ for all metalloproteins whose values of Δ*Z*_ET_ and *Z* have been measured. * Denotes that the change in charge is expressed per 1e^−^.

With redox reactions involving metalloproteins, it remains analytically challenging to determine the outer sphere reorganization of protons, solvent, co-solvent and buffer, and the adjustment of side chain charge. This analytical gap makes it hard to answer “how” and “why” metalloproteins do or do not regulate their *Z* in response to ET (or couple proton(s) to electron transfer). Protein charge ladders can – at least – inform us about the net change in charged groups in protein ET reactions. The results of this study continue to demonstrate the utility of this technique for probing ET and PCET processes in metalloproteins. The current results for 4Cu-SOD1 adds another data point to the small but growing trend of Δ*Z*_ET_ and protein *Z* for metalloproteins (Fig. 9). The results suggest that: (i) proteins engaging in PCET can have wide ranges of net negative charge (from −5 to −10); (ii) proteins engaging in PCET and 2PCET are typically more negatively charged than proteins such as cytochrome *c* that engage in proton uncoupled, single ET. This trend points towards functional links between the net charge of metalloproteins and the type of redox reactions they perform *in vivo*.

## George Whitesides tribute by Bryan F. Shaw

True story: George Whitesides paid for my wedding tux. In full disclosure, George bought the tuxedo for me to wear at “table one” when he was awarded the Priestley medal. I was a post-doc in the lab and we had drawn straws to see who would represent the lab at the ceremony. Buying an old tux was cheaper than renting a newer one. At the Priestley dinner, I sat between George's advisor, John D. Roberts and Robert's wife, Edith. After George left the table to receive the award, I couldn’t help myself. “Professor Roberts,” I asked, “what was George like as a grad student?” Professor Roberts – who won the Priestley two decades earlier – said in his enthusiastic tenor, “Well, you see, with George it was like I was the graduate student and he was the graduate advisor.” Feeling out of league, I changed the subject to marriage advice. Professor Roberts shared his unique wisdom. I wore that same tux a few months later when my fiancée Lizz and I were married. Soon after, our lives would take a tragic turn. Our first child, Noah, was born with aggressive tumors in both of his eyes (bilateral retinoblastoma). I remember leaving my desk in George's lab to meet Lizz at the ophthalmologist because “something is wrong with Noah's eyes.” Nothing has ever been the same. When I first told George the bad news, he consoled me and kindly asked “do you need any money?” We made it through treatment but Noah lost most of his vision. When I finally left Harvard to start my own lab at Baylor University, I modified my research focus. We started working on devices to detect eye disorders in children, and then on assistive technology to help students with blindness learn science. The tools that we make have helped children with eye disorders receive early diagnoses, and have helped them be included in science. Much of the courage to dare to do these different, un-chemical things, came from watching George and the amazing members of his lab. Seventeen years later, I am still married and Noah is now 16. Thank you George, for caring about everything and everyone, all the time, as much as you do. Happy 85th birthday!

## Author contributions

MG: conceptualization, formal analysis, investigation, visualization, writing – original draft. MJG-P: data curation, investigation, writing – original draft. JCK: verification. CMD: visualization TJL: investigation. BFS: conceptualization, funding acquisition, methodology, resources, supervision, writing original draft. All authors contributed to writing, review and editing.

## Data availability

The BASH script used for the computational analysis is provided as ESI‡ in the following GitHub repository: https://github.com/Mag14011/CuCuSOD1_SI_CompMethods.

## Conflicts of interest

The authors declare no conflict of interest.

## Supplementary Material

CP-026-D4CP01989J-s001
