# Supplementary material for: Proton-coupled electron transfer at a mis-metalated zinc site detected with protein charge ladders
Source: Phys Chem Chem Phys. 2024 Aug 28;26(35):22870–81. doi: 10.1039/d4cp01989j (PMC11350471; doi:10.1039/d4cp01989j)
Supplement: CP-026-D4CP01989J-s001 [file CP-026-D4CP01989J-s001.pdf]

Supplementary Information (SI) for Physical Chemistry Chemical Physics.
This journal is © the Owner Societies 2024

Supporting Information

Proton-coupled electron transfer at a mis-metalated zinc site detected with protein charge ladders.

Mayte Gonzalez, Matthew J. Guberman-Pfeffer, Jordan C. Koone, Chad M. Dashnaw, Travis J. Lato, Bryan F. Shaw*

Department of Chemistry and Biochemistry, Baylor University, Waco, TX

*To whom correspondence should be addressed: bryan_shaw@baylor.edu

Key Words: PCET, superoxide dismutase 1, charge regulation, net charge, copper

Table S1. Metal equivalents of copper per dimeric SOD1, as determined via ICP-MS. Fewer than 0.1 equivalents of metal present per dimer classified protein as "metal free". All experiments were performed in excess copper. The buffer used for capillary electrophoresis was also tested for copper.

pH	Zn: apo WT SOD1 dimer	Cu: apo WT SOD1 dimer	Cu: WT metallated SOD1 dimer	Buffer
5.6	0.01	0.04	4.74	-0.03
7.4	-0.05	0.02	4.94	-0.10
8.3	0.01	0.04	4.76	0.02

Table S2. Computed pK_as before and after reducing the Cu ion in the canonical Zn-binding site of SOD1.

		Subunit #1			Subunit #2		
Residue ID	Residue Name	$pK_{a,ox}$	$pK_{a,red}$	$\Delta pK_{a,(red.-ox.)}$	$pK_{a,ox}$	$pK_{a,red}$	$\Delta pK_{a,(red.-ox.)}$
3	Lys	11.7	11.0	-0.7	11.6	11.5	-0.1
9	Lys	9.4	9.4	0.0	9.5	9.4	-0.1
11	Asp	3.8	3.9	0.0	3.8	3.8	0.0
21	Glu	4.7	4.7	0.0	4.6	4.3	-0.3
23	Lys	10.2	10.3	0.1	10.2	10.3	0.1
24	Glu	4.6	4.6	0.0	4.5	4.6	0.1
30	Lys	10.3	10.3	0.1	10.4	10.3	-0.1
36	Lys	9.9	9.8	-0.1	10.0	9.9	-0.1
40	Glu	3.9	3.9	0.0	3.6	4.4	0.9
43	His	11.0	11.5	0.5	11.4	11.4	0.0
49	Glu	5.1	5.3	0.2	5.1	5.2	0.1
52	Asp	1.5	1.5	0.0	1.4	1.4	-0.1
70	Lys	10.5	10.1	-0.4	9.9	10.2	0.3
75	Lys	10.5	10.5	0.0	10.4	10.5	0.0
76	Asp	3.3	3.3	0.0	3.3	3.4	0.2
77	Glu	4.5	4.4	-0.1	4.4	4.4	0.0
78	Glu	3.7	3.7	0.0	3.8	3.9	0.0
90	Asp	3.0	3.1	0.2	2.7	3.1	0.4
91	Lys	10.9	10.9	0.0	10.9	10.8	0.0
92	Asp	5.1	5.4	0.3	4.9	5.2	0.2
96	Asp	4.3	4.6	0.3	4.6	4.4	-0.2
100	Glu	4.0	4.2	0.2	4.3	4.3	0.0
101	Asp	-1.8	-2.4	-0.7	-3.0	-1.6	1.4
109	Asp	3.4	3.4	0.0	3.3	3.6	0.3
110	His	7.1	6.7	-0.3	6.9	7.0	0.2
121	Glu	3.7	3.9	0.2	3.7	3.8	0.1
122	Lys	10.2	10.2	-0.1	10.2	10.2	0.1
124	Asp	-1.8	0.8	2.6	-2.3	0.6	2.9
125	Asp	2.5	3.5	1.0	2.6	2.2	-0.4
128	Lys	10.5	10.7	0.2	10.6	10.7	0.1
132	Glu	3.6	3.8	0.2	3.8	3.9	0.0
133	Glu	4.6	4.6	0.0	4.7	4.9	0.2
136	Lys	10.3	10.3	0.0	10.3	10.3	0.0

Table S3. Summary of classical molecular dynamics simulation conditions and timescales.

Redox State	Zn-Site Bonded Ligands	Zn-Site Non-Bonded Ligands	Simulated pH	Simulated Time (ns)
Cu^{+1} Cu^{+2}	His-63, His-71, His-80, Asp-83		-4, -2, 0, 2, 4, 6, 8, 10, 12	24 ns at each pH
Cu^{+1} Cu^{+1}	His-63, His-71, His-80, Asp-83		-4, -2, 0, 2, 4, 6, 8, 10, 12	24 ns at each pH
Cu^{+1} Cu^{+1}	His-71, His-80, Asp-83	His-63 (N_ϵ protonated)		314
Cu^{+1} Cu^{+1}	His-71, His-80, Asp-83	His-63 (N_ϵ and N_δ protonated)		313
Cu^{+1} Cu^{+1}	His-63, His-80, Asp-83	His-71 (N_ϵ protonated)		308
Cu^{+1} Cu^{+1}	His-63, His-80, Asp-83	His-71 (N_ϵ and N_δ protonated)		291
Cu^{+1} Cu^{+1}	His-63, His-71, Asp-83	His-80 (N_ϵ protonated)		292
Cu^{+1} Cu^{+1}	His-63, His-71, Asp-83	His-80 (N_ϵ and N_δ protonated)		382
Cu^{+1} Cu^{+1}	His-63, His-71, His-80	Asp-83 (unprotonated)		300
Cu^{+1} Cu^{+1}	His-63, His-71, His-80	Asp-83 (protonated)		331
